# Identifying and Measuring Administrative Harms Experienced by Hospitalists and Administrative Leaders

**DOI:** 10.1001/jamainternmed.2024.1890

**Published:** 2024-06-24

**Authors:** Marisha Burden, Gopi Astik, Andrew Auerbach, Greg Bowling, Kirsten N. Kangelaris, Angela Keniston, Aveena Kochar, Luci K. Leykum, Anne S. Linker, Matthew Sakumoto, Kendall Rogers, Natalie Schwatka, Sara Westergaard

**Affiliations:** 1 Division of Hospital Medicine, University of Colorado School of Medicine, Aurora; 2 Division of Hospital Medicine, Northwestern University Feinberg School of Medicine, Chicago, Illinois; 3 Division of Hospital Medicine, University of California, San Francisco; 4 Division of Hospital Medicine, University of Texas Health, San Antonio,; 5 Division of Hospital Medicine, Icahn School of Medicine at Mount Sinai Hospital, New York, New York; 6 Medicine Service, South Texas Veterans Health Care System, Department of Veterans Affairs, San Antonio; 7 Division of Hospital Medicine, University of New Mexico, Albuquerque; 8 Center for Health, Work & Environment, Department of Environmental & Occupational Health, Colorado School of Public Health, University of Colorado Anschutz Medical Campus, Aurora; 9 Division of Hospital Medicine, University of Wisconsin School of Medicine and Public Health, Madison; 10 Department of Medicine, Dell Medical School, The University of Texas at Austin

## Abstract

**Importance:**

Administrative harm (AH), defined as the adverse consequences of administrative decisions within health care that impact work structure, processes, and programs, is pervasive in medicine, yet poorly understood and described.

**Objective:**

To explore common AHs experienced by hospitalist clinicians and administrative leaders, understand the challenges that exist in identifying and measuring AH, and identify potential approaches to mitigate AH.

**Design, Setting, and Participants:**

A qualitative study using a mixed-methods approach with a 12-question survey and semistructured virtual focus groups was held on June 13 and August 11, 2023. Rapid qualitative methods including templated summaries and matrix analysis were applied. The participants included 2 consortiums comprising hospitalist clinicians, researchers, administrative leaders, and members of a patient and family advisory council.

**Main Outcomes and Measures:**

Quantitative data from the survey on specific aspects of experiences related to AH were collected. Focus groups were conducted using a semistructured focus group guide. Themes and subthemes were identified.

**Results:**

Forty-one individuals from 32 different organizations participated in the focus groups, with 32 participants (78%) responding to a brief survey. Survey participants included physicians (91%), administrative professionals (6%), an advanced practice clinician (3%), and those in leadership roles (44%), with participants able to select more than one role. Only 6% of participants were familiar with the term *administrative harm* to a great extent, 100% felt that collaboration between administrators and clinicians is crucial for reducing AH, and 81% had personally participated in a decision that led to AH to some degree. Three main themes were identified: (1) AH is pervasive and comes from all levels of leadership, and the phenomenon was felt to be widespread and arose from multiple sources within health care systems; (2) organizations lack mechanisms for identification, measurement, and feedback, and these challenges stem from a lack of psychological safety, workplace cultures, and ambiguity in who owns a decision; and (3) organizational pressures were recognized as contributors to AHs. Many ideas were proposed as solutions.

**Conclusions and Relevance:**

The findings of this study suggest that AH is widespread with wide-reaching impact, yet organizations do not have mechanisms to identify or address it.

## Key Points

**Question:**

What are common administrative harms (AHs), defined as the adverse consequences of administrative decisions within health care, as experienced by hospitalist clinicians and leaders?

**Findings:**

In this qualitative study using a mixed-methods approach, AH was noted to be pervasive and come from all levels of leadership with wide-reaching impact, that organizations lack mechanisms for identification, measurement, and feedback related to AH, and that organizational pressures drive administrative harms.

**Meaning:**

The findings of this research underscore the potential for organizations to implement structures and processes aimed at measuring and addressing AH to enhance organizational decision-making.

## Teaser

This qualitative study using a mixed-methods approach examines the identification of and proposed measures to address administrative harms.

## Introduction

Administrative harm (AH), a term initially characterized by Chang and Liang as a “quiet epidemic”^1^ and later termed formally by O’Donnell,^2^ is a longstanding problem in health care, yet it is often unnamed and overlooked. Administrative harm can be defined as the adverse consequences of administrative decisions within health care and directly influences patient care and outcomes, professional practice, and organizational efficiencies regardless of employment setting.

In the context of an increase in clinician moral distress^3^ and burnout,^4^ examining the role of AH in clinicians’ daily work experiences is vital. While the literature on these topics does not refer to AH specifically, it is suggestive of the presence of this phenomenon.^5^ Clinicians report discomfort with organizational decision-making influenced by regulations, financial incentives, external pressures, time constraints, uncertainty, and the growing influence of corporate interests in health care.^3^ Administrative decisions driven by profits often prioritize short-term gains while neglecting the longer-term negative consequences. As a result, patients are directly harmed^6^ and the health care workforce experiences moral injury and disillusionment.^3,7^ These influences, subsequent decision-making, and resulting injuries affect the culture and integrity of health care organizations as well as the broader health care system.^7,8^

In clinical settings, assessing the impacts of clinician behaviors, such as communication and medical decision-making, in errors and harms has become commonplace. However, administrative decision-making and the resulting AH has remained largely unexplored. We embarked on a qualitative study using a mixed-methods approach to explore the experiences of hospitalist clinicians and leaders with AHs, understand challenges that may exist in identifying and measuring AH, and understand potential approaches to mitigate AH.

## Methods

### Study Design

Because AH is an emerging concept, we used an exploratory mixed-methods approach.^9,10^ This involved an embedded design consisting of a brief survey and focus groups conducted concurrently. This design allowed us to systematically assess focus group participants’ familiarity and experiences of AH using survey results with semistructured interviews facilitating rich dialogue and examples regarding participant experiences. We used a rapid qualitative approach to data analysis.^11,12^ A rapid approach includes near real-time, team-based data analysis to identify themes. This rapid approach allowed us to address a time-sensitive and emerging area of research, laying the foundation for action-oriented steps to better understand and address AH. The institutional review board at the University of Colorado approved this study. The protocol is available in Supplement 1. Email advertisements were sent across participating organizations. Verbal consent was obtained at the beginning of each session. Participants were not offered incentives to participate. This study followed the framework for the Consolidated Criteria for Reporting Qualitative Research (COREQ) reporting guideline.^13^

### Context and Setting

This study was conducted within 2 hospitalist communities. Participants for this study included hospitalist clinicians, leaders, researchers, and patient and family advisory council members who are a part of the HOMERuN Research Network^14^ and the Society of Hospital Medicine Academic Leaders Special Interest group forum, both of which are national organizations. The focus groups were held during preset meeting times of the 2 groups. Multiple focus groups were held concurrently.

### Participants and Sampling Strategies

Forty-one individuals participated in the focus groups, with 32 participating in the survey. There were 10 total focus groups. Inclusion criteria allowed clinicians, administrative leaders (clinical or otherwise), researchers, and any other participants that attend the HOMERuN^14^ and Special Interest Group forums. The exclusion criterion was refusal to participate; no individuals refused. The focus groups were predominantly physicians, with 4 of the 10 focus groups having nonphysician members.

### Study Team

Several members of the team (M.B., G.A., A.A., G.B., K.N.K., A. Keniston, L.K.L., A.S.L., N.S., S.W.) had previous experience with qualitative methods and have held or hold administrative director–type roles (M.B., G.A., G.B., A. Keniston, L.K.L.). In some instances, researchers may have had professional knowledge of or previous working relationships with the participants given the national networking that occurs in professional societies, such as the Society of Hospital Medicine and HOMERuN.

### Data Collection

Because the term *administrative harm* has received limited attention in the health care literature and there is an absence of validated questions or tools to assess AH in this context, we used an artificial intelligence tool (ChatGPT, versions 3.5 and 4; Open AI) to assist in generating and refining the preliminary survey and focus group guide questions in conjunction with the expertise and guidance from the research team that oversaw the editing and finalization of the questions (eMethods in Supplement 2). A link to the REDCap^15^ survey was placed in the virtual platform chat (Zoom; Qumu Corp) during the chat session (embedded design). The survey was 12 questions long and took less than 5 minutes to complete.

Data were collected on June 13, 2023 (4 concurrent focus groups), and August 11, 2023 (6 concurrent focus groups) using semistructured focus groups. Before the sessions commenced, participants were provided an article describing AH,^2^ and a 5-minute presentation was delivered at the beginning of the sessions, given many participants were unfamiliar with the term administrative harm. Some of us (M.B., G.A., G.B., K.N.K., A.S.L., M.S., K.R., N.S., S.W.) served as moderators for the focus groups. At the end of the focus groups, an at-large session was held for moderators to share the learnings in a deidentified fashion, which allowed for participants to add any thoughts to the discussion. Focus group sessions were held via video conferencing and were recorded, with each breakout focus group session lasting 31 minutes for all focus groups (ie, focus groups were held concurrently). The audio recordings and field notes were used for analysis rather than verbatim transcriptions. Brief REDCap^15^ surveys were completed during the time allotted for the focus groups.

### Analysis

In alignment with rapid qualitative methods, templated summaries and matrix analysis were used^12,16,17,18,19^ instead of line-by-line coding methods. The research team summarized (ie, abstracted) focus group content directly from the audio recordings, which is a data consolidation technique used with rapid qualitative methods.^11,19,20^ Templated summary structure was guided by the central questions from the focus group guide^12,19^ (Supplement 1). To ensure uniformity in content, style, and organization, the research team collectively summarized 1 focus group session and discussed the summary to standardize and calibrate the approach. The remaining focus groups were then assigned to individual team members to repeat the process (M.B., K.N.K., A. Keniston, A. Kochar, M.S., N.S., S.W.). One research team member (M.B.) reviewed all the videos and each of the templated summaries to ensure consistency across summaries.

Following the templated summary process, a matrix was created (Excel; Microsoft Corp) to display the summarized data. Each team member (M.B., G.A., G.B., K.N.K., A. Keniston, A. Kochar, A.S.L., M.S., K.R., N.S., S.W.) followed a structured process of analyzing the contents of the matrices. We conducted thematic analysis using a mixed deductive (ie, starting with the central questions and domains from the focus group guide) followed by an inductive (ie, an exploratory approach for a topic that has had limited study to date in health care) approach. Study team members discussed findings iteratively cross-checking against the matrix, memos, field notes, and focus group recordings until consensus was reached on the themes and subthemes. Quantitative analysis was performed using SAS Enterprise Guide 8.3 (SAS Institute Inc).

### Data Integration, Reflexivity, and Data Validation Techniques

Triangulation was used to integrate the survey findings with the qualitative findings to provide a more in-depth understanding of the results.^21,22^ The research team regularly practiced reflexivity,^23^ acknowledging and discussing personal influences on data interpretation and analysis through multiple reflective sessions, both before and after each of the focus group dates. Twenty-nine percent of the participants were sent a summary of the overall results and given the opportunity to comment on the findings. No substantial changes were suggested.

## Results

**Figure 1.**
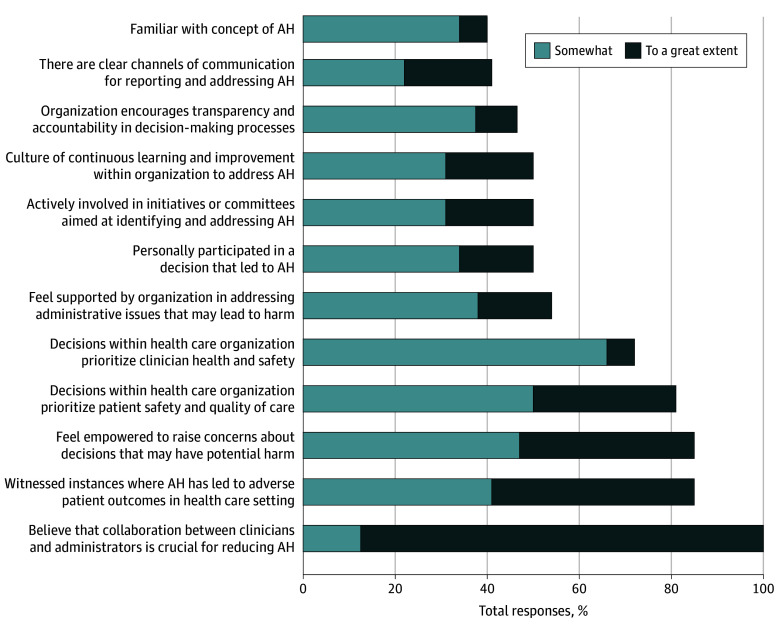
Survey Results for 32 Participants Who Completed the Survey AH indicates administrative harm.

Ten semistructured virtual focus groups were held with 41 individuals from 32 different organizations on June 13 and August 11, 2023. All but one organization were within the US. Thirty-two participants completed the survey (78% response rate) (Table 1). Survey participants included physicians (91%), administrative professionals (6%), an advanced practice clinician (3%), and those in leadership roles (44%), with participants able to select more than one role. Survey results are provided in Figure 1 and in eTable 1 in Supplement 2. Three key themes emerged along with subthemes and are presented in Table 2 with survey findings integrated into the qualitative themes.

**Table 1.** Survey Demographic Characteristics^a^

Characteristic	Participants (n = 32), No. (%)
Role^b^	
Advanced practice clinician	1 (3)
Administrative professional	2 (6)
Leader	14 (44)
Physician	29 (91)
Other^c^	1 (3)
Years in practice, mean (SD)	14 (6)
% Clinical full-time equivalent, mean (SD)	53 (24)

^a^ Given embedded design, survey responses are from those who participated in the focus groups and completed a survey during the session.

^b^ Participants could select more than one role.

^c^ Medical education.

**Table 2.** Themes and Subthemes

Theme and subtheme	Representative quotation
**Theme 1: AH is pervasive and comes from all levels of leadership with wide-reaching impact.**	
Subtheme 1.1: AH comes from all levels of leadership, both within an organization and beyond.	“…[M]onths into the pandemic, we had gotten very good at all of the things to do to deal with surges. Someone in the risk management department at the health system said wait a minute we do not have evidence that the physicians and APPs have completed a required training like they do for hand hygiene, and sharps, injury prevention, etc. And so, there was an urgent email that went out basically saying you have 72 hours to complete this 1 hour and 15 minutes training on COVID safety or your credentials will be suspended. This created tremendous stress as you might imagine both given the time, urgency, which felt undoable for a lot of people who were really burned out and struggling and given that everybody felt we were quite good at preventing ourselves from getting COVID...” (Focus group 2) “It [regarding recent CMS changes over APP split shared billing] is an administrative change coming from a high level that is not going to improve patient care for us at all. It just adds burden…I try to drop notes early in the day to communicate the plan of care. How can I do a time-based note if I don’t know what time is going into the patient care throughout the entirety of the day because I have to answer pages and go back to the bedside? I don’t know a feasible way to do time-based billing on an inpatient model without committing some element of fraud or only doing bills midday. There is patient harm in doing the latter and then you have potential provider harm in the former in that you are being disingenuous.” (Focus group 3)
Subtheme 1.2: AH negatively impacts patients, the workforce, and organizations.	“A great example of administrative harm is the theme of…when we have complex systems and we require physicians to work themselves through that system because we don’t have the capacity to make the system better.” (Focus group 1) “Generally, on staffing issues, there’s a lot in that bucket…There’s just not enough hands-on deck to safely transition these patients to the outpatient setting. It’s hard to measure that directly because sometimes we don’t document exactly why patients end up staying longer but I feel if we had more hands-on deck that would happen less often.” (Focus group 4) “We had a new CEO come in and we already have a strained emergency room, like I am assuming most everyone does, but he really wanted to ramp up accepting of transfers into the hospital which just further burdened and everybody wants to please the new CEO so now we have a pipeline of people coming from states and states away to just sit in the emergency room in the hall…” (Focus group 7) “As a person who does admin trying to advocate for resources and support to keep our team census …at a reasonable level because we believe that results in … a sustainable workforce, but the administrative oversight that I’m working with, they think, well, you’re actually reducing from the bottom line and if we don’t have the bottom line then we can’t do this.” (Focus group 9)
**Theme 2: Organizations lack mechanisms for identification, measurement, and feedback related to AH.**	
Subtheme 2.1: AH is challenging to measure.	“I don’t think it is easily measured and I don’t think the people who are in charge of metrics want it measured. So, those 2 things in and of themselves make it challenging and if you keep the worker bees at the frontline busy enough, they don’t have time.” (Focus group 1) “…I have never even heard of somebody measuring from the administrative or hospital side that this kind of harm even exists and most of the time what happens is we are asked to do a task, and that is not a question… the only thing when folks get upset, there are so many physicians getting angry down the road, that this is not feasible we cannot do this, this is stressful, then the administrators will listen, maybe this is not what we think…then they will stop but that is like a last resort.” (Focus group 7) “I think this question gets to the heart of the issue because I don’t think we have a great way of tracking these outcomes…we just have accepted this is the system that we work in. At our hospital, we tried to get a dashboard for how many days patients spend as medically ready for discharge. We wanted to say hey we want to have a checkbox that we can check when our patient is medically ready for discharge and then we can count the number of days it takes to discharge. We were told that is a terrible idea because the insurance companies will come in and deny payment for everything after that checkmark...” (Focus group 8)
Subtheme 2.2: There is a lack of leadership responsibility and accountability in AH.	“The decision around post-acute care, we are transitioning to more insurance companies requiring authorization to get into skilled nursing. It has ‘BOOM’ instantly added 3 days to our length of stay since the pandemic. It's 3 more days of hospital-acquired infections or other harms. It is ridiculous. We have absolutely no leverage over insurance companies, and they ought to be held accountable.” (Focus group 3) “We couldn’t get Echos on the weekend… and the way that administration solved that problem was if someone really needed an Echo there is always a cardiology fellow who can just come in and work extra hours for free and do that. I have seen many examples where inadequate staffing is tolerated by administration because, you know, we know that thing needs to be done and we will get it done somehow and you can always get an underpaid trainee to do the work. Administration really has no particular reason to change that if patients are still getting the care they need.” (Focus group 9)
**Theme 3: Organizational pressures drive AH.**	
Subtheme 3.1. There are characteristics of decisions that result in AH.	“We actually paid a very high-cost consulting company to tell us that we needed to eliminate our administrative time and staff to 18 to 20 patients per hospitalist and cut vacant positions and the list goes on and on…we were paying consultants to tell us how to do hospitalist work, most of whom were these new grads that said they were a hospitalist…They were being paid a ton of money to tell us how to do our work and then on top of it they were basically trying to create a burn and churn program…” (Focus group 2) “There is a proximity effect – if you go back to patient safety principles – Swiss cheese – sort of the sharp end. A lot of the administrative structures and functions sit pretty far from the sharp end of care – and so right now they are invisible like we have been talking about. If we were to simply ask the question to our organization – what are the categories to which an administrative decision might flow downstream, I don’t even know that we could name categories much less the metrics.” (Focus group 2) “Everybody has an incentive to say, ‘new project is helping’ and there’s not a lot of incentive to say, ‘whoa let’s slow down maybe there’s unintended consequences.’” (Focus group 4)
Subtheme 3.2: AH stems from communication and decision-making silos.	“Some of the issue is that ultimately a lot of the safety events involve administration and when we have administrative issues they somehow think that it is not an administrative issue it is just the way it is and just as that article that was sent out – it is a budget cut…it is just a restructuring and they don’t see it through the filter that it is really an issue with them…you guys have to find work arounds or how to work through it or whatever they identify the issue as and not really something they have done.” (Focus group 2) “It was a business decision made by business folks without any clinical input…Where does that come from? [Is] folks not understanding, not being at bedside, not engaging frontline workers, and not having a clinical background and not reaching out to understand if ‘I make this decision, yes it fixes my budget, but what ramifications does it cause downstream?’. That’s where I think a lot of these decisions happen because people are making them in a silo...” (Focus group 7)

Abbreviations: AH, administrative harm; APP, advanced practice provider; CEO, chief executive officer; CMS, Centers for Medicaid & Medicare Services; Echo, echocardiogram.

### Theme 1: AH Is Pervasive and Comes From All Levels of Leadership With Wide-Reaching Impact

#### Subtheme 1.1: AH Comes From All Levels of Leadership, Both Within an Organization and Beyond

Participants perceived that AH is pervasive and stems from all levels of leadership (including administrators, clinicians, and anyone else with decision-making authority), leading to extensive impact throughout the health care system. Despite the pervasiveness of AH, most participants did not know there was a term to describe these effects of administrative decisions. Survey findings supported this perception, as only 6% of participants were familiar with the term to a great extent.

#### Subtheme 1.2: AH Negatively Impacts Patients, the Workforce, and Organizations

Administrative harm was noted to negatively affect patients, the workforce, and organizations. Administrative harm was perceived to encompass diverse entities, including logistics in accomplishing everyday patient care tasks (eg, ordering radiographic imaging and paperwork for patients) to clinician group or health system level issues (eg, initiatives such as early discharge, decisions on staffing, and availability of resources) to national initiatives (eg, policy decisions, payer reimbursement). eTable 2 in Supplement 2 provides more examples. Regarding the workforce, AH was felt to lead to daily workarounds, requiring the workforce to bypass established protocols to provide patients the care they need. Administrative harm was noted to erode workforce trust, lead to moral injury, and even lead to turnover. While some participants raised questions about labeling it as AH if the harm was solely experienced at the workforce level, the prevailing thought was that these harms are pervasive and often have cascading effects from the workforce to patients. This underscored the variation in how AH was perceived, ranging from an inevitable byproduct of decisions (an acceptable status quo) to recognizing it as a symptom of a dysfunctional management system that can be rectified (reversible or avoidable harm). Administrative harm was often felt to be graver when it was a root cause of clinical harm to patients; 85% of participants noted that AH had led to patient harm at least somewhat or to a great extent.

### Theme 2: Organizations Lack Mechanisms for Identification, Measurement, and Feedback Related to AH

#### Subtheme 2.1: AH Is Challenging to Measure

Administrative harm was uniformly noted to be challenging to measure and it was felt that there are often perverse incentives to not measure it. Current leadership structures often lack ownership of downstream adverse effects, in part because of decisional distancing (ie, effects of decisions are apparent much further downstream). Many participants noted that this distance, in addition to power dynamics, further limit feedback on the impact of decisions. Fear of personal retribution and of unintended consequences for what may be perceived as complaining limited the feedback loop. In contrast to measuring and reporting clinical harms, many participants noted no such mechanism to report AH. While 44% of the participants reported that administrative decisions had led to adverse patient outcomes to a great extent, only 38% of participants felt that they were empowered to speak up and raise concerns.

#### Subtheme 2.2: There Is a Lack of Leadership Responsibility and Accountability in AH

Because many policies are implemented without clarity as to the leader or decision-maker, assigning ownership to decisions that cause AH was felt to be difficult. Although organizations frequently use dashboards or scorecards to visually display important metrics, such as length of stay, readmissions, or profit/loss statements, participants perceived that such tools are not typically applied to administrative decisions or the decision-making process.

### Theme 3: Organizational Pressures Drive AH

#### Subtheme 3.1. There Are Characteristics of Decisions That Result in AH

The need to act combined with a lack of organizational memory was noted to contribute to a cycle of recurring AHs. Participants noted that organizational leaders often perceive any action as a positive change, regardless of whether it results in the desired outcome. The characteristics of decision-making that were felt to lead to AH included making decisions in the setting of pressing issues, not including the voice of the customer (eg, patients, workforce), failing to incorporate balancing measures, neglecting to evaluate the potential for harm adequately before acting, and overcorrection of an issue.

#### Subtheme 3.2: AH Stems From Communication and Decision-Making Silos

Communication and decision-making silos were felt to exacerbate AH by impeding the flow of information among teams and by preventing collaborative problem-solving for issues that affect the frontline workforce. There was concern about an us vs them narrative around business professionals vs clinicians, with participants noting that collaborations with administrators and clinicians are crucial (100%, somewhat to great extent). Many examples centered on attributing harm from others’ decision-making; however, 81% of the participants acknowledged their own involvement in contributing to AH. External third-party consultants were frequently mentioned as sources of AH, and multiple participants perceived this was due to their lack of firsthand experience or understanding of the problems they were hired to address.

### Solutions to Mitigate AH

Many ideas were proposed as solutions. Exemplar quotations and connections to survey questions are reported in Table 3.

**Table 3.** Solutions to Prevent and Address Administrative Harm

Solution	Actionable tactic	Respondents answering “to a great extent,” % (n = 32)	Representative quotation
Develop a definition and understanding of administrative harm.	Acknowledge and understand AH and its significance. Understand the scope of AH can be patients, workforce, and organizational outcomes. Quantify and understand costs of AH. Develop a conceptual framework for AH.	Familiar with AH, 6%. AH led to adverse patient outcomes, 44%.	“It’s a new term to me, so it hasn’t been one that I’ve been thinking about, but I almost think it should, could be part of any root-cause analysis and I would almost ask it as an implication. What were the AHs associated with this case? Because undoubtedly there are some. And I think it’s hard to see it. It’s why the more we talk, the more that are coming to mind because we are conditioned…you know you get acclimated to your workload...I like the term, it certainly resonates.” (focus group 3) “It [the term admin harm] also made me feel guilty because I have inflicted it on many people, you know? So that’s a good reaction, right? And there’s a visceral reaction to ‘Yes, I’ve done that’, you know?” (focus group 10)
Foster a collaborative culture and psychological safety.	Embrace a culture of psychological safety to encourage open discussions and problem-solving. Avoid us vs them mentality. Promote transparency. Develop feedback mechanisms. Open communication. Relationship building across disciplines. Aspire to administrative health by fostering partnerships and collaborative decision-making. Know key stakeholders (patients, workforce, organizations). Leader rounding and familiarity with work being done at the ground level. Involving patients and caregivers in decision-making. Interprofessional collaboration. Build trust.	Collaboration between clinicians and administrators is crucial to reducing AH, 88%. Feel empowered to speak up and raise concerns about AH, 38%.	“I think it’s making it not an us versus them. We need physicians who understand the business of health care…I need administrators who understand clinical care… But I think that shared mental models and shared governance, shared leadership, and mutual understandings is really important.” (Focus group 8) “…there is some fear of retribution or ill feelings if you are exposing things that happen in a complex situation…with the complex nature of health care, I don’t think any one person really knows how one decision affects other parts of the hospital, it’s just there’s so many layers to it, and so I think outside of being part of that conversation, I think transparency is an important piece too.” (Focus group 9) “What’s the opposite of administrative harm? … administrative health? What you’re really talking about is partnership and collaboration. You’re trying to shift from some unilateral decision-making with unclear incentives and unclear drivers to something that’s more transparent and collaborative.” (Focus group 10)
Develop structure and processes to support optimal decision-making.	Develop time-out framework for decision-making. Pause and confirm understanding of the goals of the decision. Accountability for decision-making. Develop guiding principles and values for informed and inclusive decision-making. Proactive approach to evaluating decision-making (look backs). Promote transparency and follow-up on outcomes. Involve more junior team members in the decision-making. More clinicians with business training at the decision-making table and more administrative partners with an understanding of clinical work. Shared mental models and collaborative decision-making. Pilots with follow-up measures with commitment to reevaluate or change strategy if not achieving results. Getting comfortable with saying no and setting boundaries. Sufficient resources to address the problem.	Organization encourages transparency and accountability in administrative decision-making, 9%. Feel supported in addressing administrative issues that may lead to harm, 16%.	“There are university mandates, trainings you can do, HIPAA training, that is all fine and good, but I wonder if we said, classifying ‘For whom at the bequest of whom are these things done’, it might be important framing as well… None of us are ever going to be bothered by a patient, none of us have ever said ‘Don’t call me.’” (Focus group 1) “I think it’s an uncommon practice unfortunately for everybody to assess the impact of their change universally whether it’s starting or stopping a thing even though we know it’s best practice… I think there’s not enough true assessment of the impact of change and as a subset of that, there’s definitely not enough assessment of the harm of changes and I think that harm comes in patient harm, efficiency harm, workload, and well-being harm...” (Focus group 4) “I always think of how could you be more proactive instead of reactive, so if there’s a way to cultivate, encourage, and invest in proactive ideas to avoid administrative harm...” (Focus group 9)
Develop measurement and data strategies for administrative harm.	Utilize qualitative and quantitative evaluations. Conceptualize into buckets, something more tangible. Measure outcomes such as impacts on the workforce, patients, and organizations. Include balancing measures. Define and measure value that a third-party consultant brings. Measure costs of AH. Track outcomes of meetings (value add vs not value add meetings).	Administrative decisions prioritize patient safety and quality, 31%. Administrative decisions prioritize clinician health and safety, 6%.	“Defining the value of an external consult, and why people choose to go with an external consultant, rather than doing things internally. There are a lot of politics around those decisions that I don’t entirely understand but the impact is we spend a lot of health care dollars on consultants.” (Focus group 3) “We need to quantify the health care costs associated with the administrative burden: how many excess days patients spend in the hospital, how many readmissions are due to medication denials, etc.” (Focus group 8) “I think of viewing evaluation as a scarce resource. So, big decisions, like big changes that have the potential for harm, I think a commitment to following up on what the downstream consequences of that decision and reevaluating if that’s the right decision…” (Focus group 10)
Develop reporting and learning systems.	Implement an incident reporting system to track and learn from AH and organizational decision-making. Develop multidisciplinary review processes. Evidence-based decisions. Utilizing quality improvement tools. Utilize patient stories.	Actively involved in initiatives or committees aimed at identifying and addressing AH, 19%. Clear channels of communication for reporting and addressing AH, 19%. Culture of continuous learning and improvement to address AH, 19%.	“… if you go deep into the Lean Six Sigma play book, there are these 2 components before we do any change—one is the voice of the process—so what is the process, the measuring processes and its outcomes and the other is the voice of the customers which I think in a simplified fashion we have to think of as the patients and families, but the other customer is anyone who uses the process and you go and you sit down with them and say how might a change impact your workflow and really gather that voice of the customer information. That often brings a bunch of insights about where things are going to go off the rails with a certain change. I feel that we have stopped doing the voice of the customer because we don’t have time, we are too smart, there’s not enough money but it turns out that is an important step.” (Focus group 2) “…I will be talking about it [AH] with others because I do think we have an opportunity to put something in place like the recommendation that was made or alluded to that there is no place to report AH.” (Focus group 6)

Abbreviations: AH, administrative harm; CMO, chief medical officer; HIPAA, Health Insurance Portability and Accountability Act.

## Discussion

**Figure 2.**
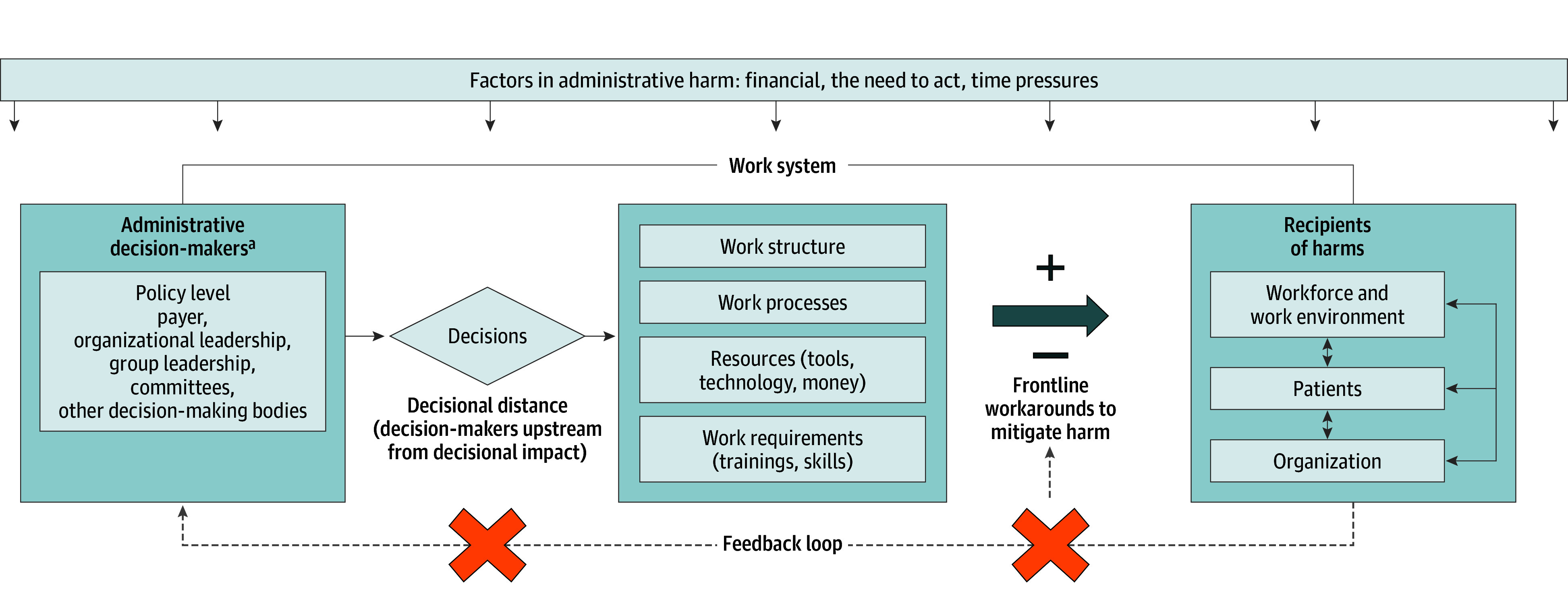
Conceptual Model for Administrative Harm There are multiple factors in administrative harm that impact the overall work system and ultimately the recipients of the harms. Administrative decision-makers often operate far upstream from the consequences of their decisions. These decisions impact work structure, processes, resources, and work requirements, which can then positively or negatively impact the workforce, patients, and organizations. The frontline workforce often resorts to workarounds to minimize harm. Because of the distance of decision-makers from the impact of their decision, lack of clarity where high-level decisions may originate from, and a lack of psychological safety, there is often the lack of a feedback loop. This inhibits effective organizational learning. ^a^Administrative decision-makers are inclusive of, but not limited to, administrative professionals including nonclinicians and clinicians. The term is inclusive of anyone with decision-making authority or influence regardless of role, title, or degree.

There are several important implications that come from this work. First, it is challenging to address an issue when it has no name. Participants readily identified examples of AH and noted its pervasiveness yet lacked a term for it. Next, just as organizations have built robust structures and processes to assess decision-making in the clinical setting, there are similar opportunities to address AH building on several of the ideas presented by O’Donnell.^2^ Participants noted specific characteristics of decisions that lead to AHs and many opportunities to address AHs. We developed a conceptual model for AH (Figure 2) from the findings.

Our results underscore the lack of organizational processes to identify, measure, and provide feedback on administrative decision-making. In clinical medicine, there are multiple mechanisms to measure and track errors and provide individual and organizational learning through quality and safety reporting systems^24^ and morbidity and mortality conferences.^2,25,26,27^ However, system-level changes are routinely undertaken without an understanding of their potential effects on clinical outcomes. The organizational blind spots for AH coupled with power dynamics inhibit organizational learning, presenting potential opportunities for improvement. This highlights the opportunity for collaboration and shared understanding between those who are making decisions and those affected by decisions.

The use of third-party consultants as a specific contributor to AH repeatedly surfaced in our focus groups and was perceived in all instances described to have had wide-reaching negative impact. Harms stemming from third-party consultants have been published in the mainstream media,^28^ which has depicted their influence as invisible, yet ubiquitous, suggesting that such influence has contributed to harms as large as the opioid epidemic and safety lapses at major corporations. There are also likely third-party collaborations that are useful. Understanding when and how these partnerships can be helpful will be paramount.

There was a perceived lack of collaboration in administrative decisions and the feeling of an us vs them stance. This is problematic in the context of changes in the health care ecosystem that make physician employment in larger health care organizations the norm. The shift from physician-owned practices to employment has been rapid. As of 2022, nearly 50% of physicians were employed.^29^ These evolving workforce trends may also contribute to the growing interest among physicians in considering and pursuing unionization as a means to introduce checks and balances into organizational decision-making and leverage collective bargaining.^30^ While the concept of unionization did not emerge as a central theme, it was mentioned in relation to the moral crisis clinicians are facing and the increasing trend of unionization of housestaff.

Consideration of the broader health care landscape in which AH operates is important. In the US, health care spending surpasses that of any other country, with administrative roles emerging as one of the fastest-growing sectors,^2,31^ with a 28% projected increase in medical and health services manager jobs from 2022 to 2032 compared with physician projected growth of 3%.^32^ A related change is the increase in private equity investment in health care. While investors with a profit motivation have long been involved in health care, the recent increase has been substantial, particularly with regard to physician practices.^33^ The association between investors’ acquisition of health care delivery organizations and negative clinical outcomes has been reported.^6^ These findings highlight the importance of understanding the interplay of administrative decision-making by health care leaders for both care recipients and the health care workforce.

In addition, contemplating AH prompts the consideration for what is administrative success in a health care system where profit often takes precedence. Organizations must critically evaluate whether their decision-making leads to tangible gains, financial and otherwise. Adopting a more holistic approach to organizational decision-making that includes financial sustainability, workforce health and safety, and patient needs will likely yield much higher gains over the long run.

### Strengths and Limitations

To our knowledge, this study is one of the first to characterize AH in the health care setting and offer potential research-based solutions. The work included participants from many different organizations, although predominantly from academic organizations. The findings thus may not be transferable to other settings. We used rapid qualitative methods that are designed to deliver focused, actionable data within a condensed timeframe.^11,12^ Given this methodologic approach, we did not use line-by-line coding. As a result, some nuanced findings may have not been fully explored. However, we used multiple approaches to ensure thoroughness of the data (ie, member checking, report outs, researchers embedded in the context/setting, and triangulation). While patient and family advisory council members participated in the focus groups, they do not appear to have filled out the survey, and thus their perspective is missing. In addition, the participants were predominantly physicians, which could impact the findings. As this study’s goals were exploratory in nature, additional examination in other health care worker groups will be needed.

## Conclusions

In this qualitative study using a mixed-methods approach, AH was found to be pervasive and have wide-reaching impact, and was challenging to identify and measure. However, participants identified many opportunities to address it.

## Notes

Supplement 1.
Trial Protocol

Supplement 2. **eMethods.** Detailed Methods **eTable 1.** Survey Results, N = 32 **eTable 2.** Examples of Sources of Administrative Harm

Supplement 3.
Data Sharing Statement
