# Supplementary material for: Identifying and Measuring Administrative Harms Experienced by Hospitalists and Administrative Leaders
Source: JAMA Intern Med. 2024 Jun 24;184(9):1014–23. doi: 10.1001/jamainternmed.2024.1890 (PMC11197021; doi:10.1001/jamainternmed.2024.1890)
Supplement: Supplement 1. — Trial Protocol [file jamainternmed-e241890-s001.pdf]

SUPPLEMENT 1: PROTOCOL

Project Title: The Administrative Harm Project: Collaborative Approaches to Reducing Administrative Errors in Healthcare Systems, A Qualitative Study of Frontline Clinicians and Leaders

Principal Investigator: Marisha Burden, MD

Background:

Administrative harm, also referred to as "adminogenic injury",[1] has been a longstanding and pervasive problem in healthcare. Administrative harm encompasses decisions made by healthcare leaders that can have adverse effects on the healthcare workforce, patients, and organizational outcomes.[1,2] Administrative harm can arise from legislative decisions and the actions or inaction of organizational-level leaders. Chang and Liang previously described the "quiet epidemic"[2] of potentially harmful administrative decisions that were burdening clinicians and patients.[1,2] The decisions described were noted to hinder patient access to clinically indicated medications and testing, leading to a term called "management malpractice." Examples of administrative harm includes a wide range of decisions, including those that may result in insufficient staffing or lack of access to care for critically important services. To date, our work has focused on building evidence-based practices to determine optimal workloads that drive worker, patient, and organizational outcomes. Our work has highlighted the harms that can occur when decisions are not thoughtfully executed including strain on the workforce and patient harm.[3,4] These decisions may also result in worse organizational outcomes such as reduced throughput, rework, and financial outcomes.[5] While Morbidity and Mortality Conferences and patient safety reviews are commonplace to review care decisions that have led to patient harm, there is limited evaluation of administrative decisions or retrospective analysis to enhance decision-making. Unlike clinical harm, administrative harm remains largely unexplored. To address this issue, we aim to utilize rapid qualitative methods to explore common administrative harms as experienced by frontline hospitalists and hospitalist leaders, how we might measure administrative harm, and explore potential processes for ongoing organizational learning and reduction of administrative harm. We have chosen this population given the dynamic nature of inpatient care and the high level of illness for hospitalized people that makes changes to processes of care higher stakes in terms of outcomes. Hospitalists are also now involved in the care of hospitalized persons across health conditions and service lines and makes this population well suited to provide broadly relevant information to the topic. The ultimate goal of this work is to foster collaboration among healthcare professionals, including clinicians and administrators, to build better work environments and processes to minimize administrative harm, and construct systems of accountability for continuous improvement.

Specific Aims:

(1) Explore common administrative harms experienced by frontline hospitalist clinicians and hospitalist leaders.
(2) Understand challenges that exist in identifying and measuring administrative harm in healthcare.
(3) Understand potential approaches to mitigate administrative harm and improve patient, workforce, and organizational outcomes.

Preliminary Studies: To date, there have been only two perspective pieces covering this topic and we are unaware of work aimed at identifying administrative harm or work aimed at developing an understanding to measure and mitigate it.

Methods

Study design: This study will utilize rapid qualitative methods, including virtual focus groups and report-out structures, to collect and analyze data from hospitalist clinicians and hospitalist leaders. The study will incorporate a phenomenological approach to understand the experience of frontline hospitalists and hospitalist leaders with administrative harm.

Description of participant selection: Participants will be recruited from two national networks of healthcare professionals in the United States including the HOMERuN Research Network[6] and participants in a special interest group forum from the Society of Hospital Medicine (Chiefs Special Interest Group).

Inclusion criteria: Participants will include clinicians, administrators, and other healthcare professionals that attend the HOMERuN[6] and Special Interest Group forums. These two forums already have existing meeting structures to discuss pertinent issues including conducting focus groups. In advance of the already scheduled monthly meetings, an email notice will go out to potential participants letting them know of the agenda. Anyone attending the call can participate. Verbal consent will be conducted at the beginning of the session.

Exclusion criteria: Refusal to participate.

Data collection: We will follow methods as adapted by our team.[7] Data will be collected through virtual focus groups using Zoom. There will be two sessions with multiple breakout groups. Participants will be asked open-ended questions about their experiences with administrative harm in healthcare settings. Focus groups will be audio-recorded, and transcripts will be created using Zoom or Microsoft transcription services. We will also conduct a brief survey with individuals who agree to participate in the focus groups. Survey data will be anonymous and collected in REDCap.

Interviewer/facilitators: All interviewers will have been trained in conducting focus groups and will be provided the interview guide in advance. Moderators will not actively participate in the discussion; however, will guide the discussion based upon prompts. Field notes will also be taken during the sessions.

Interview guide: The interview guide was based upon literature review as well as the lived experience of clinicians, researchers, and clinician leaders. The guide was refined through group discussion. See guide for details. We utilized the AHRQ Culture of Safety questions[8] and Stanford professional fulfillment index[9] as reference points to develop the questions.

Interviews: We anticipate the interviews will last 45 minutes to 60 minutes (the duration of the already scheduled meeting formats). Because of the nature of the focus groups and already scheduled meetings, we will not be assessing for data saturation.

Because there are overlaps with individuals that participate in the two entities (Society of Hospital Medicine and HOMERuN), there may be individuals who attend both focus groups. If an individual attends a second focus group, we will try to place those individuals in a group whereby they are with others that attended the other session. The interview guide will be adapted for this group given they will have already reflected and provided insights into the responses. Because the purpose of these two venues is information sharing with a goal of program improvement and quality improvement, we will not discourage participation in the second event.

Data analysis: Templated summaries and matrix analysis[10-12] will be used to analyze the data.[7] Templated summaries will be organized based on the central questions from the focus group guide. The matrix tool will be used to display the summarized data, and matrix analysis will be conducted to identify recurring concepts and patterns. The analysis phase will involve each team member following a structured process of analyzing the contents of the matrices and making notes as they go. Team members will use questions such as “What did you notice? Any surprises? Any variation? Repetitive ideas? Any conflicting ideas?” to guide their analysis.[7,12] Thematic analysis will be conducted using a mixed inductive and deductive method at the semantic level.[13] Member checking will be conducted.[14] This will entail report outs after the focus groups, a session summary that will be emailed to the group at large, as well as individual outreach. Materials emailed for member checking will not include any identifying information.

Research team and reflexivity: The research team will consist of clinicians and researchers who have experience with rapid qualitative methods. All team members who participate in the sessions will be trained (if not already experienced in the methodology) to follow the rapid qualitative methods via interview guides and guided analysis and discussions. Reflexivity will be applied by having team members regularly check their assumptions and biases throughout the study using a structured team-based approach.

Methodological orientation and theory: The qualitative methodological approach with which this project most closely aligns is phenomenology.[15] The interpretive framework of pragmatism will be applied to identify solutions for real-world challenges with an appreciation of the contextual diversity in which research and quality improvement can take place.[15]

Study outcomes: The study will produce recommendations for measuring, evaluating, and addressing administrative harm in healthcare settings.

Description, risks, and justification of procedures and data collection tools:
Human Subjects Protection: The principal risk to subjects is a potential harm resulting from confidentiality. To reduce this risk, interview and focus group audio files and transcriptions will be stored in a password-protected folder on a secure password protected server accessible by study staff only. All identifiers will be removed from any transcripts prior to analysis. There are some limitations to using the method. Rapid qualitative analysis yields preliminary results[12] that provide a high-level overview of the data and may aid in understanding future areas of focus. Due to our use of concurrent focus groups, data saturation will not be assessed, making it possible key themes were missed. To mitigate this, summaries will be sent to the research network and special interest group members and members will be asked to provide feedback to ensure the findings are as representative as possible (i.e., member checking). The quicker pace must be balanced with the need for more detailed analysis, recognizing that more nuanced findings could be overlooked.[16]

Potential Scientific Problems, Expected Results, and Implications: The results of this project should help our research team to understand experiences with administrative harm, how to measure administrative harm, and how to mitigate administrative harm. The information will be summarized for each of the forums and shared (summarized content only) in the spirit of continuous learning.

Funding Source: None

Timeline

Activities	May	Jun	Jul	Aug	Sep	Oct	Nov
IRB approval							
Focus Groups							
Analysis							
Manuscript/dissemination efforts							

References

1. O'Donnell WJ. Reducing Administrative Harm in Medicine - Clinicians and Administrators Together. *N Engl J Med*. Jun 23 2022;386(25):2429-2432. doi:10.1056/NEJMms2202174

2. Chang HJ, Liang MH. A piece of my mind. The quiet epidemic. *JAMA*. Nov 2 2011;306(17):1843-4. doi:10.1001/jama.2011.1587

3. Arogyaswamy S, Vukovic N, Keniston A, et al. The Impact of Hospital Capacity Strain: a Qualitative Analysis of Experience and Solutions at 13 Academic Medical Centers. *J Gen Intern Med*. Dec 13 2021;doi:10.1007/s11606-021-07106-8

4. Burden M, Patel M, Kissler M, Harry E, Keniston A. Measuring and driving hospitalist value: Expanding beyond wRVUs. *J Hosp Med*. Jun 2 2022;doi:10.1002/jhm.12849

5. Burden M, Keniston A, Gundareddy VP, et al. Discharge in the a.m.: A randomized controlled trial of physician rounding styles to improve hospital throughput and length of stay. *J Hosp Med*. Apr 2023;18(4):302-315. doi:10.1002/jhm.13060

6. Auerbach AD, Patel MS, Metlay JP, et al. The Hospital Medicine Reengineering Network (HOMERuN): a learning organization focused on improving hospital care. *Acad Med*. Mar 2014;89(3):415-20. doi:10.1097/ACM.0000000000000139

7. Keniston A, McBeth L, Astik G, et al. Practical Applications of Rapid Qualitative Analysis for Operations, Quality Improvement, and Research in Dynamically Changing Hospital Environments. *Jt Comm J Qual Patient Saf*. Feb 2023;49(2):98-104. doi:10.1016/j.jcjq.2022.11.003

8. Survey on Patient Safety Culture Version 2.0: User’s Guide. (Prepared by Westat, under Contract No. HHSP233201500026I/HHSP23337004T). Rockville, MD: Agency for Healthcare Research

and Quality; June 2021. AHRQ Publication No. 19(21)-0076.
https://www.ahrq.gov/sops/surveys/hospital/index.html.

9. Trockel M, Bohman B, Lesure E, et al. A Brief Instrument to Assess Both Burnout and Professional Fulfillment in Physicians: Reliability and Validity, Including Correlation with Self-Reported Medical Errors, in a Sample of Resident and Practicing Physicians. *Acad Psychiatry*. Feb 2018;42(1):11-24. doi:10.1007/s40596-017-0849-3

10. Hamilton A. Qualitative Methods in Rapid Turn-Around Health Services Research. https://www.hsrd.research.va.gov/for_researchers/cyber_seminars/archives/780-notes.pdf Accessed May 1, 2021.

11. Zuchowski JL, Chrystal JG, Hamilton AB, et al. Coordinating Care Across Health Care Systems for Veterans With Gynecologic Malignancies: A Qualitative Analysis. *Med Care*. Jul 2017;55 Suppl 7 Suppl 1:S53-S60. doi:10.1097/MLR.0000000000000737

12. Moreau J. Rapid Qualitative Methods. Colorado Pragmatic Research in Health Conference. August 12, 2020. Aurora, Colorado.

13. Braun V, Clarke V. Using thematic analysis in psychology. *Qualitative Research in Psychology*. 2006/01/01 2006;3(2):77-101. doi:10.1191/1478088706qp063oa

14. Birt L, Scott S, Cavers D, Campbell C, Walter F. Member Checking: A Tool to Enhance Trustworthiness or Merely a Nod to Validation? *Qual Health Res*. Nov 2016;26(13):1802-1811. doi:10.1177/1049732316654870

15. Creswell J and Poth C. Qualitative Inquiry and Research Design: Choosing Among Five Approaches. Fourth Edition. SAGE Publications. January 2017.

16. Taylor B, Henshall C, Kenyon S, Litchfield I, Greenfield S. Can rapid approaches to qualitative analysis deliver timely, valid findings to clinical leaders? A mixed methods study comparing rapid and thematic analysis. *BMJ Open*. Oct 8 2018;8(10):e019993. doi:10.1136/bmjopen-2017-019993

Administrative Harm Project

Moderator Guide

June, 2023

5-minute whole group presentation on the topic and poll questions:

Lead Marisha Burden, MD

Hello. My name is Marisha Burden. Thank you all for taking the time to join today. We appreciate your willingness to participate! We're here today to talk about administrative harm.

There is a link to a short survey in the chat. Please be sure to complete the survey before the session is over.

Participants will be asked if they participated in the focus group on administrative harm in June 2023 through the Society of Hospital Medicine Chief's Special Interest Group: (yes/no).

Survey questions *All rated on a 1-4 scale, to a great extent, somewhat, very little, not at all*

1. Before today, I was familiar with the concept of administrative harm.
2. The administrative decisions within my healthcare organization prioritize patient safety and quality of care.
3. The administrative decisions within my healthcare organization prioritize clinician health and safety.
4. I feel supported by my organization in addressing administrative issues that may lead to harm.
5. There are clear channels of communication for reporting and addressing administrative harm in my healthcare setting.
6. I believe that collaboration between clinicians and administrators is crucial for reducing administrative harm in healthcare.
7. I am actively involved in initiatives or committees aimed at identifying and addressing administrative harm in my healthcare organization.
8. My organization encourages transparency and accountability in administrative decision-making processes.
9. I have witnessed instances where administrative harm has led to adverse patient outcomes in my healthcare setting.
10. I have personally participated in a decision that led to administrative harm.
11. I feel empowered to speak up and raise concerns about administrative decisions that may have potential harm.
12. There is a culture of continuous learning and improvement within my organization to address administrative harm.

Demographics questions:

1. How many years have you been in practice?
2. What is your role? (check all that apply)

APP
Physician

PhD
Leader
Administrative professional
Patient representative
Other: Free text
3. Clinical effort (enter percent effort) (i.e., the percent effort you work clinically: E.g., 70% cFTE would mean you spend 70% of your time in clinical care)
4. Institution Name: Free text
5. Institution City, State: Free text

[Short presentation on the topic of administrative harm.]

Your participation is strictly voluntary, and all of your responses will be completely anonymous. It is important that what is said here, stays here so that we can create a safe environment to speak freely without judgement or negative repercussions. We will record our discussion today as we want to be able to capture everything that is said, but we will not be reporting on one specific person or disclose any information that will identify a person, company or specific location in the transcript or final reports.

Does everyone agree to these ground rules? Do you have any questions? Are you ready to get started?

30-minute breakouts to discuss focus group questions:

Lead Moderators

Introductions

Disclaimer: We are going to have a small group discussion on administrative harm. We would like to audio record these groups to summarize. We will disseminate this information back to the network, and potentially use the information as parts of future summaries or presentations. We will NOT use the recordings for any other purpose, send them to anyone else, or quote them by name. You can choose not to participate in the small group discussions, and if you decide to participate, do not have to speak and can leave at any time.

In the chat box, introduce yourself. Name, institution, and current role and responsibilities.

Interview Guide

Question 1:
Describe a specific instance of administrative harm that you or a colleague experienced.

Prompt: How did this harm affect patient care, clinicians, or other outcomes?
Prompt: What factors contributed to this incident of administrative harm?
Prompt: Where does administrative harm come from? (i.e., certain people, types of worker?)

Question 2:
What challenges exist in identifying and measuring administrative harm in healthcare?

Prompt: Describe how your organization identifies and measures administrative harm.
Prompt: How could we effectively measure the impact of administrative harm on patient outcomes and healthcare costs?
Prompt: What tools or strategies do you think would be most effective in identifying administrative harm?
Prompt: What tools or strategies do you think would be most effective in preventing administrative harm?

Question 3:
How can healthcare professionals work together to mitigate administrative harm and improve patient and clinician outcomes?

Prompt: What strategies have you seen implemented/or that could be implemented in healthcare settings to address administrative harm?

10–15-minute whole group report out and discussion

Lead: Marisha Burden, MD

- Insert brief survey link, into chat box.
- Ask each group moderator to report out on conversation
- Allow for questions and discussions
- Wrap up and thank you

Interview guide for participants that participated in the first session (i.e., follow up session).

Interview Guide

Question 1:
Now that you have had the opportunity to discuss and explore administrative harm during our last session, how has your view of that discussion or administrative harm changed?

Prompt: How did the last session impact how you think about administrative harm?
Prompt: What types of administrative harm have you seen recently?
Prompt: Where are the sources or origins of this administrative harm?
Prompt: What is the significance of administrative harm? How has your view changed?

Question 2:
What do you think of the term ‘administrative harm’?

Prompt: What were your initial reactions to the term ‘administrative harm’?
Prompt: How have those reactions changed (if they have) since we last met?
Prompt: How does this term compare to other terms used to indicate harm?

Prompt: What is the best way to describe this phenomenon (currently termed 'administrative harm')?

Question 3:
What strategies have you thought about to implement or measure administrative harm?

Prompt: What are the challenges with those strategies?
Prompt: How do you think others will perceive the strategies?
Prompt: What do you think the impact of those strategies might be?
