# Supplementary material for: Identifying and Measuring Administrative Harms Experienced by Hospitalists and Administrative Leaders
Source: JAMA Intern Med. 2024 Jun 24;184(9):1014–23. doi: 10.1001/jamainternmed.2024.1890 (PMC11197021; doi:10.1001/jamainternmed.2024.1890)
Supplement: Supplement 2. — eMethods. Detailed Methods eTable 1. Survey Results, N = 32 eTable 2. Examples of Sources of Administrative Harm [file jamainternmed-e241890-s002.pdf]

Supplementary Online Content

Burden M, Astik G, Auerbach A, et al. Identifying and measuring administrative harms experienced by hospitalists and administrative leaders. *JAMA Intern Med*. Published online June 24, 2024. doi:10.1001/jamainternmed.2024.1890

eMethods. Detailed Methods

eTable 1. Survey Results, N = 32

eTable 2. Examples of Sources of Administrative Harm

This supplementary material has been provided by the authors to give readers additional information about their work.

© 2024 Burden M et al. *JAMA Internal Medicine*.

eMethods. Detailed Methods

Context/setting

HOMERuN is a consortium of over 50 academic hospital medicine programs focused on improving quality and implementation science. The Society of Hospital Medicine (SHM) Academic Leaders Special Interest Group primarily consists of leaders, typically Division or Section heads of academic groups across the country.

Study team and reflexivity:

Several members of the team (AA, AKe, GA, GB, KK, LL, MB, NS, SW) had previous experience with qualitative methods. AA, AKo, GA, GB, KK, KR, LL, MB, SW are physicians. AKe has a PhD in Clinical Science, NS has a PhD in Occupational Health and Safety. AKe, GA, GB, LL, and MB have each held or hold administrative director type roles. MB oversaw all aspects of the project. AL, GA, GB, KK, KR, MB, MS, NS, SW served as moderators for the focus groups. AKe, AKo, KK, MB, MS, NS, SW participated in the templated summary process. AKe, AKo, AL, GA, GB, KK, KR, MB, MS, NS, SW participated in the matrix analysis.

In some instances, the researchers may have had professional knowledge or previous working relationships with the participants given the national networking that occurs in professional societies such as SHM and HOMERuN. In order to ensure a safe space for sharing, participants could request to be reassigned focus groups (e.g., we attempted to ensure that there were no supervisors serving as moderators and similarly if a participant felt there was any other conflict of interest, they could exit out of their assigned focus group and request another one). Because of the national reputations of the researchers, some of the participants could have been aware of their previous work. The researchers on this project all have an interest in building better workplace practices. We do not believe there were inherent biases influencing the work, however, to mitigate any possibility, the team met weekly to

© 2024 Burden M et al. *JAMA Internal Medicine*.

discuss the project, findings, observations, and how the study team's own experiences could bias the results.

Procedures

During the sessions, administrative support team members divided participants up into the various focus groups with an average of four participants per group (ranging from two to six). Priority was made to ensure no supervisors were moderators. As there were participants who were members in both SHM and HOMERuN, it resulted in four individuals attending both sessions, and we did not discourage their participation in the second event given the primary focus of these events is information sharing across the respective organizations and our goal was to gain as deep of understanding of AH as possible. However, for survey results, only the first response is included in the results. These individuals who participated twice (4 individuals) were offered to join a specific focus group as a follow up to the first session. Two individuals selected this option. The interview guide was modified for this group (see protocol for details). No participants refused to participate in the study, however, one did have Zoom connectivity issues and wasn't able to fully participate.

Data Collection: Development of Survey and Qualitative Questions.

Because the term AH has received limited attention in the healthcare literature and there is an absence of validated questions or tools to assess AH in this context, we utilized the ChatGPT tool by Open AI systems (versions 3.5 and 4) to assist in generating and refining the preliminary survey and focus group guide questions in conjunction with the expertise and guidance from the research team which oversaw the editing and finalization of the questions. Specifically, we utilized a variety of prompts and inputs to generate the preliminary survey and qualitative questions. We have provided examples of the prompts utilized. Because edits to the questions were iterative between ChatGPT and the research team, we do not have each version of the respective questions.

© 2024 Burden M et al. *JAMA Internal Medicine*.

Inputs: Previous qualitative protocols and rapid qualitative methods developed by our team; intro paragraph on administrative harm

Command: Here is an example of a template that I want to follow. For the next prompt I will share with you the new topic. [our previous protocol entered]

Command: and then I want to use these methods [our previous methods entered]

Inputs:

Preliminary protocol with objectives entered for a workshop on a similar topic.

Command [following the above]:

Help me to develop three qualitative questions with a few prompts.

Output: Sure, here are three possible qualitative questions with prompts:

1. What are some examples of administrative harm that you have experienced or witnessed in healthcare settings?

- Can you describe a specific instance of administrative harm that you or a colleague experienced?

- How did this harm affect patient care or outcomes?

- In your opinion, what factors contributed to this incident of administrative harm?

2. How can healthcare professionals work together to mitigate administrative harm and improve patient-centered care?

- What strategies have you seen implemented in healthcare settings to address administrative harm?

- In your opinion, what role do administrators and clinicians play in mitigating administrative harm?

- How can we better involve patients and families in efforts to reduce administrative harm and promote patient-centered care?

3. What challenges exist in identifying and measuring administrative harm in healthcare, and how can we address these challenges?

© 2024 Burden M et al. *JAMA Internal Medicine*.

- What are some common barriers to identifying and addressing administrative harm in healthcare settings?
- How can we effectively measure the impact of administrative harm on patient outcomes and healthcare costs?
- What tools or strategies do you think would be most effective in identifying and preventing administrative harm?

Next, our members of our research team then edited and discussed making iterative revisions with the team and ChatGPT. The final guide is provided in the protocol.

Survey questions:

Input: Here is a protocol [protocol inserted] I plan to submit with qualitative questions. I also want to ask a brief survey.

I want to mirror these questions however make them pertinent to administrative harm/ this project. Suggestions for questions? Survey questions

All rated on a 1-5, strongly disagree to strongly agree, scale

1. My organization demonstrates commitment to employee safety and health.
2. My hospitalist team leader urges me to assume workplace health and safety responsibilities on my own.
3. My hospitalist team members behave in a way that displays a commitment to health and safety at work.
4. My hospitalist team members and I spend time discussing our organization's purpose, goals, and expectations for workplace health and safety.
5. I proactively develop and make suggestions for workplace health and safety issues that may influence our hospitalist team.
6. I see myself as a workplace health and safety leader.

© 2024 Burden M et al. *JAMA Internal Medicine*.

Source for questions came from: Schwatka NV, Keniston A, Astik G, et al. Hospitalist Shared Leadership for Safety, Health, and Well-Being at Work: United States, 2022–2023. *Am J Public Health*. Feb 2024;114(S2):162-166. doi:10.2105/AJPH.2024.307573

Output:

Here are some additional survey questions that can be pertinent to the topic of administrative harm and this project:

1. The administrative decisions within my healthcare organization prioritize patient safety and quality of care.
2. I feel supported by my organization in addressing administrative issues that may lead to harm.
3. There are clear channels of communication for reporting and addressing administrative harm in my healthcare setting.
4. I am aware of the processes and resources available to mitigate administrative harm within my organization.
5. I believe that collaboration between clinicians and administrators is crucial for reducing administrative harm in healthcare.
6. I am actively involved in initiatives or committees aimed at identifying and addressing administrative harm in my healthcare organization.
7. My organization encourages transparency and accountability in administrative decision-making processes.
8. I have witnessed instances where administrative harm has led to adverse patient outcomes in my healthcare setting.
9. I feel empowered to speak up and raise concerns about administrative decisions that may have potential harm.

© 2024 Burden M et al. *JAMA Internal Medicine*.

10. There is a culture of continuous learning and improvement within my organization to address administrative harm.

Input: for the survey above - what do you think about this feedback? I would like to consider a different scale, given agreement scales suffer from acquiescence bias, perhaps something like this:

Input: are there any questions that could be removed or should be added? essentially administrative harm can happen at all levels.

Output:

The set of questions you have provided covers various aspects related to administrative harm within a healthcare organization. While all the questions are relevant, here are a few suggestions for improvement:

1. Consider rephrasing Question 1 to make it more specific and measurable. For example:
    - "I understand the specific types of administrative harm that can occur within my healthcare organization."
2. Add a question about the organization's commitment to addressing and preventing administrative harm at all levels. For example:
    - "My healthcare organization actively promotes a culture that prevents administrative harm at all levels."
3. Consider adding a question about the availability and accessibility of training or education related to administrative harm prevention. For example:
    - "My healthcare organization provides adequate training and education to help prevent administrative harm."
4. Include a question about the responsiveness of the organization in addressing reported incidents of administrative harm. For example:

© 2024 Burden M et al. *JAMA Internal Medicine*.

- "When I report incidents of administrative harm, my healthcare organization takes prompt action to address the issue."

These suggestions aim to enhance the comprehensiveness of the survey by incorporating additional dimensions related to administrative harm prevention and response. However, the final decision on which questions to include or exclude should be based on the specific context and goals of your survey.

The research team in conjunction with the use of the ChatGPT tool then conducted iterative discussions and edits. Questions were not pilot tested beyond the individuals on the study team. The survey also included demographics questions. Individuals were not provided definitions of the various roles offered in the survey and participants were able to self-identify. Leaders was considered a broad category. Participants could choose multiple options. The final survey is provided in the protocol.

Data Collection: Additional Survey Procedures

A link to the REDCap survey was placed in the Zoom chat towards the end of the sessions to gauge participant's experiences with AH given the research team felt that it was unlikely that the participants would be familiar with the term AH (embedded mixed methods design).

Additional Details on Analysis and Findings: AKe, AKo, KK, MB, MS, NS, SW participated in the templated summary process. AKe, AKo, AL, GA, GB, KK, KR, MB, MS, NS, SW participated in the matrix analysis. To analyze the matrix, research team members looked across rows, down columns, and then in a zig zag pattern to identify recurring concepts and patterns. In keeping with rapid qualitative methods, line-by-line coding was not conducted.

© 2024 Burden M et al. *JAMA Internal Medicine*.

Additional Data Integration Details:

Triangulation was used to integrate the survey findings with the qualitative findings to provide a more in depth understanding. The research team discussed the findings to identify any convergent or divergent themes across the quantitative and qualitative data.

Data validation techniques

Multiple measures were taken to ensure that the data were as comprehensive as possible including conducting focus groups with two different entities and multiple member checking processes. Due to the confidential nature of the comments and study approach, we did not return transcriptions to participants for comment or correction.

© 2024 Burden M et al. *JAMA Internal Medicine*.

eTable 1. Survey results, N = 32

	Not at all N (%)	Very little N (%)	Somewhat N (%)	To a great extent N (%)
Before today, I was familiar with the concept of administrative harm.	10 (31%)	9 (28%)	11 (34%)	2 (6%)
The administrative decisions within my healthcare organization prioritize patient safety and quality of care.	0 (0%)	6 (19%)	16 (50%)	10 (31%)
The administrative decisions within my healthcare organization prioritize clinician health and safety.	2 (6%)	7 (22%)	21 (66%)	2 (6%)
I feel supported by my organization in addressing administrative issues that may lead to harm.	2 (6%)	13 (41%)	12 (38%)	5 (16%)
There are clear channels of communication for reporting and addressing administrative harm in my healthcare setting.	9 (28%)	10 (31%)	7 (22%)	6 (19%)
I believe that collaboration between clinicians and administrators is crucial for reducing administrative harm in healthcare.	0 (0%)	0 (0%)	4 (13%)	28 (88%)
I am actively involved in initiatives or committees aimed at identifying and addressing administrative harm in my healthcare organization.	11 (34%)	5 (16%)	10 (31%)	6 (19%)
My organization encourages transparency and accountability in administrative decision-making processes.	5 (16%)	12 (38%)	12 (38%)	3 (9%)
I have witnessed instances where administrative harm has led to adverse patient outcomes in my healthcare setting.	3 (9%)	2 (6%)	13 (41%)	14 (44%)
I have personally participated in a decision that led to administrative harm.	6 (19%)	10 (31%)	11 (34%)	5 (16%)
I feel empowered to speak up and raise concerns about administrative decisions that may have potential harm.	2 (6%)	3 (9%)	15 (47%)	12 (38%)
There is a culture of continuous learning and improvement within my organization to address administrative harm.	6 (19%)	10 (31%)	10 (31%)	6 (19%)

© 2024 Burden M et al. *JAMA Internal Medicine*.

eTable 2. Examples of sources of administrative harm

Domains	Examples
Administrative workload	Promotion and tenure tasks; salaries not charged appropriately; over-engineered systems such as patient flow
Availability of services	Generalized staffing issues; patient care service availability at certain sites requiring increased patient transfers; lacking resources for disease specific patient teaching; delays in care secondary to administrative processes (assigning beds); lack of certain services on weekends in hospitals; delays in urgent dialysis because of needing higher level of care which can delay patient care; lack of sufficient PT/OT staff leading to lack of early mobilization and deconditioning
Communication issues	Referral systems that do not communicate
Consultants	Often focused on resource reduction and are costly
Documentation	Payor billing changes; filling out paperwork
Electronic health record	Electronic health records not merging for individual patients (creating duplicate records leading to repeat treatments and tests); clinicians not able to access records with partner organizations (inhibiting scheduling)
Email volume	Inbox effect
Employee benefits or services	Paying for parking at your place of employment
External forces (e.g., insurance companies, payors, state funding)	Payor issues driven by lack of insurance coverage; post-acute care authorization processes
Growth/expansion	System level leadership diminishing local leadership and focus at the local level which can lead to financial strain and hiring freezes to allow for growth
Ill-conceived quality improvement efforts (cost, execution)	Time based discharge initiatives (discharge before noon or other arbitrary time)
Lack of collaboration	Lack of collaboration on high level decision making; using different electronic health record systems that don’t communicate with each other
Lack of reliable data	Unclear when patients are medically ready for discharge; reliability of data shared by hospital leading to demoralization of workforce
Meeting volume	Daily huddles that may not be good use of time; unnecessary administrative meetings

© 2024 Burden M et al. *JAMA Internal Medicine*.

Overcorrection of issues	Delirium precautions protocol (such as reduced frequency of overnight checks for certain patients) eliminated and now patients with delirium woken up overnight; COVID training months into pandemic to follow human resources practice
Patient volumes	Increasing transfers into hospitals but no capacity leading to boarding patients in emergency department
Procedures and processes	Response to clinical errors (overcorrecting policies); unable to get patients to sub-acute care; not involving patients in patient-facing care documents (e.g. medical jargon in discharge summary content); clinical workflows (e.g. observation workflows, geographic cohorting)
Safety event	Staffing issues leading to unsafe practices and inefficiencies
Staffing	Travel nurses to plug holes
Training	Operating room fire training for all; redundant training; ineffective annual training

PT/OT = physical therapy/occupational therapy

© 2024 Burden M et al. *JAMA Internal Medicine*.
