# Supplementary material for: Identifying and Measuring Administrative Harms Experienced by Hospitalists and Administrative Leaders
Source: JAMA Intern Med. 2024 Jun 24;184(9):1014–23. doi: 10.1001/jamainternmed.2024.1890 (PMC11197021; doi:10.1001/jamainternmed.2024.1890)
Supplement: Supplement 3. — Data Sharing Statement [file jamainternmed-e241890-s003.pdf]

Data Sharing Statement

Burden. Identifying and Measuring Administrative Harms Experienced by Hospitalists and Administrative Leaders. *JAMA Intern Med*. Published June 24, 2024. doi:10.1001/jamainternmed.2024.1890

Data
Data available: Yes
Data types: Deidentified participant data
How to access data: We can provide data upon request, de-identified with the following stipulations. We will make the de-identified data and associated documentation available under a data-sharing agreement that provides for: (1) a commitment to not attempting to re-identify any individual participant; (2) a commitment to securing the data using appropriate computer technology; (3) a commitment to destroying or returning the data after analyses are completed, and (4) with documentation of the appropriate regulatory approval. For each dataset produced for this project, a data dictionary will be created describing the data source, survey response rate, definitions of variables included in the dataset(s), and blank survey forms as references. Data will be delivered as CSV files to users with a data-sharing agreement. Requests can be sent to marisha.burden@cuanschutz.edu
When available: With publication

Supporting Documents
Document types: Other (please specify)
Additional Information: Protocol has been provided in the supplement.
How to access documents: Protocol has been provided in the supplement.
When available: With publication

Additional Information
Who can access the data: We can provide data upon request, de-identified with the following stipulations. We will make the de-identified data and associated documentation available under a data-sharing agreement that provides for: (1) a commitment to not attempting to re-identify any individual participant; (2) a commitment to securing the data using appropriate computer technology; (3) a commitment to destroying or returning the data after analyses are completed, and (4) with documentation of the appropriate regulatory approval. For each dataset produced for this project, a data dictionary will be created describing the data source, survey response rate, definitions of variables included in the dataset(s), and blank survey forms as references. Data will be delivered as CSV files to users with a data-sharing agreement.
Types of analyses: We can provide data upon request, de-identified with the following stipulations. We will make the de-identified data and associated documentation available under a data-sharing agreement that provides for: (1) a commitment to not attempting to re-identify any individual participant; (2) a commitment to securing the data using appropriate computer technology; (3) a commitment to destroying or returning the data after analyses are completed, and (4) with documentation of the appropriate regulatory approval. For each dataset produced for this project, a data dictionary will be created describing the data source, survey response rate, definitions of variables included in the dataset(s), and blank survey forms as references. Data will be delivered as CSV files to users with a data-sharing agreement.
Mechanisms of data availability: We can provide data upon request, de-identified with the following stipulations. We will make the de-identified data and associated documentation available under a data-sharing agreement that provides for: (1) a commitment to not attempting to re-identify any individual participant; (2) a commitment to securing the data using appropriate computer technology; (3) a commitment to destroying or returning the data after analyses are completed, and (4) with documentation of the appropriate regulatory approval. For each dataset produced for this project, a data dictionary will be created describing the data

source, survey response rate, definitions of variables included in the dataset(s), and blank survey forms as references. Data will be delivered as CSV files to users with a data-sharing agreement.

Any additional restrictions: We can provide data upon request, de-identified with the following stipulations. We will make the de-identified data and associated documentation available under a data-sharing agreement that provides for: (1) a commitment to not attempting to re-identify any individual participant; (2) a commitment to securing the data using appropriate computer technology; (3) a commitment to destroying or returning the data after analyses are completed, and (4) with documentation of the appropriate regulatory approval. For each dataset produced for this project, a data dictionary will be created describing the data source, survey response rate, definitions of variables included in the dataset(s), and blank survey forms as references. Data will be delivered as CSV files to users with a data-sharing agreement.
